# Oral Delivery of a Novel Recombinant *Streptococcus mitis* Vector Elicits Robust Vaccine Antigen-Specific Oral Mucosal and Systemic Antibody Responses and T Cell Tolerance

**DOI:** 10.1371/journal.pone.0143422

**Published:** 2015-11-30

**Authors:** Emily Xie, Abhiroop Kotha, Tracy Biaco, Nikita Sedani, Jonathan Zou, Phillip Stashenko, Margaret J. Duncan, Antonio Campos-Neto, Mark J. Cayabyab

**Affiliations:** 1 Global Infectious Disease Research Center and the Department of Immunology and Infectious Diseases, The Forsyth Institute, 245 First Street, Cambridge, Massachusetts, United States of America; 2 Department of Microbiology, The Forsyth Institute, 245 First Street, Cambridge, Massachusetts, United States of America; 3 Department of Oral Medicine, Infection and Immunity, Harvard School of Dental Medicine, Boston, Massachusetts, United States of America; Imperial College London, UNITED KINGDOM

## Abstract

The pioneer human oral commensal bacterium *Streptococcus mitis* has unique biologic features that make it an attractive mucosal vaccine or therapeutic delivery vector. *S*. *mitis* is safe as a natural persistent colonizer of the mouth, throat and nasopharynx and the oral commensal bacterium is capable of inducing mucosal antibody responses. A recombinant *S*. *mitis* (*rS*. *mitis*) that stably expresses HIV envelope protein was generated and tested in the germ-free mouse model to evaluate the potential usefulness of this vector as a mucosal vaccine against HIV. Oral vaccination led to the efficient and persistent bacterial colonization of the mouth and the induction of both salivary and systemic antibody responses. Interestingly, persistently colonized animals developed antigen-specific systemic T cell tolerance. Based on these findings we propose the use of *rS*. *mitis* vaccine vector for the induction of mucosal antibodies that will prevent the penetration of the mucosa by pathogens such as HIV. Moreover, the first demonstration of *rS*. *mitis* having the ability to elicit T cell tolerance suggest the potential use of *rS*. *mitis* as an immunotherapeutic vector to treat inflammatory, allergic and autoimmune diseases.

## Introduction

*Streptococcus mitis* is a commensal species of Gram-positive oral streptococci that inhabits the human mouth. *S*. *mitis* and other oral commensal streptococci including *S*. *salivarius* and *S*. *oralis* are among the first colonizers of the human mouth; hence, they are called pioneer oral bacteria. *S*. *mitis* colonizes hard surfaces in the oral cavity such as dental hard tissues as well as mucous membranes and *S*. *mitis* is also found in the throat and nasopharynx. *S*. *mitis* takes residence in the human oral cavity as early as 1–3 days postpartum [1] and continue to persist until other oral bacteria co-inhabit the mouth. An established adult human oral microbiota is comprised of over 600 prevalent taxa [2–4]. An extensive microbiota study showed that during infancy and adult life, *S*. *mitis* can predominate, both in prevalence and proportion of oral streptococci recovered in the mouth [5] and *S*. *mitis* was the most predominant bacterial species colonizing all oral surfaces of adults [6]. The success of *S*. *mitis* likely hinges on its ability to adapt to the oral microenvironment and evade host defenses. For example, some strains of *S*. *mitis* and other oral streptococci express IgA1 protease that cleaves host neutralizing IgA antibodies [3, 4].

Mucosal antibody responses to *S*. *mitis* are well-documented. In adult human, salivary IgA to *S*. *mitis* is present in high titers [7] and in infants, salivary antibodies are generated soon after birth. The nature of the T cell response to *S*. *mitis* is not fully understood, although, *S*. *mitis*-specific T cell clones have been isolated from human peripheral blood [8]. Certain commensal bacteria especially those found in the gut induce T cell tolerance [9] and it is possible that *S*. *mitis* and perhaps other oral commensal bacteria are also capable of inducing T cell tolerance.

The ability to safely and efficiently colonize and persist in the human mouth and other mucosal surfaces as well as the ability to induce oral mucosal antibodies are unique biologic features of *S*. *mitis* that make this pioneer bacterium a potential antigen vaccine or therapeutic delivery vehicle. The genome of *S*. *mitis* has been fully sequenced and the oral bacterium is genetically tractable for the expression of foreign antigens.

In this study, we explored *S*. *mitis’* potential as a mucosal vaccine vector for the delivery of antigens against HIV. Mucosal HIV transmission is responsible for the majority of the current estimated 34 million HIV infection globally and current vaccine strategies for HIV and other mucosal pathogens efficiently induce systemic immune responses but many do not efficiently induce mucosal immunity. Mucosal immunity is likely to play a critical role in vaccine-mediated protection against HIV. In HIV infection, the virus primarily penetrates across mucosal surfaces and viral replication in the intestinal mucosa contributes significantly to pathogenesis in adult HIV infection [10–13]. In mother-to-child transmission (MTCT), studies in an important breast milk AIDS transmission model in infant rhesus macaques suggest that HIV-1 likely penetrates oral and gut mucosal surfaces of the infant, infects intraepithelial dendritic cells and CCR5+ CD4 T cells, then disseminates to the oral buccal mucosa, tonsils, esophageal and intestinal mucosa [10]. It is argued that a preventive vaccine must induce protective mucosal antibodies at the time of transmission to prevent pathogen adherence and penetration of the epithelial surface and/or induce pathogen-specific mucosal T responses that will destroy the pathogen in infected tissues [11, 13].

We constructed a recombinant *S*. *mitis* (*rS*. *mitis*) vector prototype expressing HIV antigens and tested its ability to induce B and T cell responses in the germ-free mouse model. We found that oral inoculation with *rS*. *mitis* resulted in efficient and persistent colonization of the oral cavity of mice. Furthermore, *rS*. *mitis* markedly induced systemic and salivary vaccine antigen-specific antibody responses. Interestingly, persistent colonization by *rS*. *mitis* induced T cell non-responsiveness to antigen stimulation, which is likely due to the induction of oral T cell tolerance. These findings suggest that an *rS*. *mitis* vector prototype is capable of colonizing oral mucosal surfaces and inducing mucosal and systemic antibody responses, which argue in favor of further development of *rS*. *mitis* vaccine strategy to elicit protective mucosal and systemic antibodies against HIV and other mucosal pathogens. In addition, the ability of *rS*. *mitis* to induce systemic T cell tolerance also has clinical implication for the development of *rS*. *mitis*-based therapy to treat inflammatory, allergic and autoimmune diseases.

## Materials and Methods

### Generation of recombinant *Streptococcus mitis*

**Fig 1:**
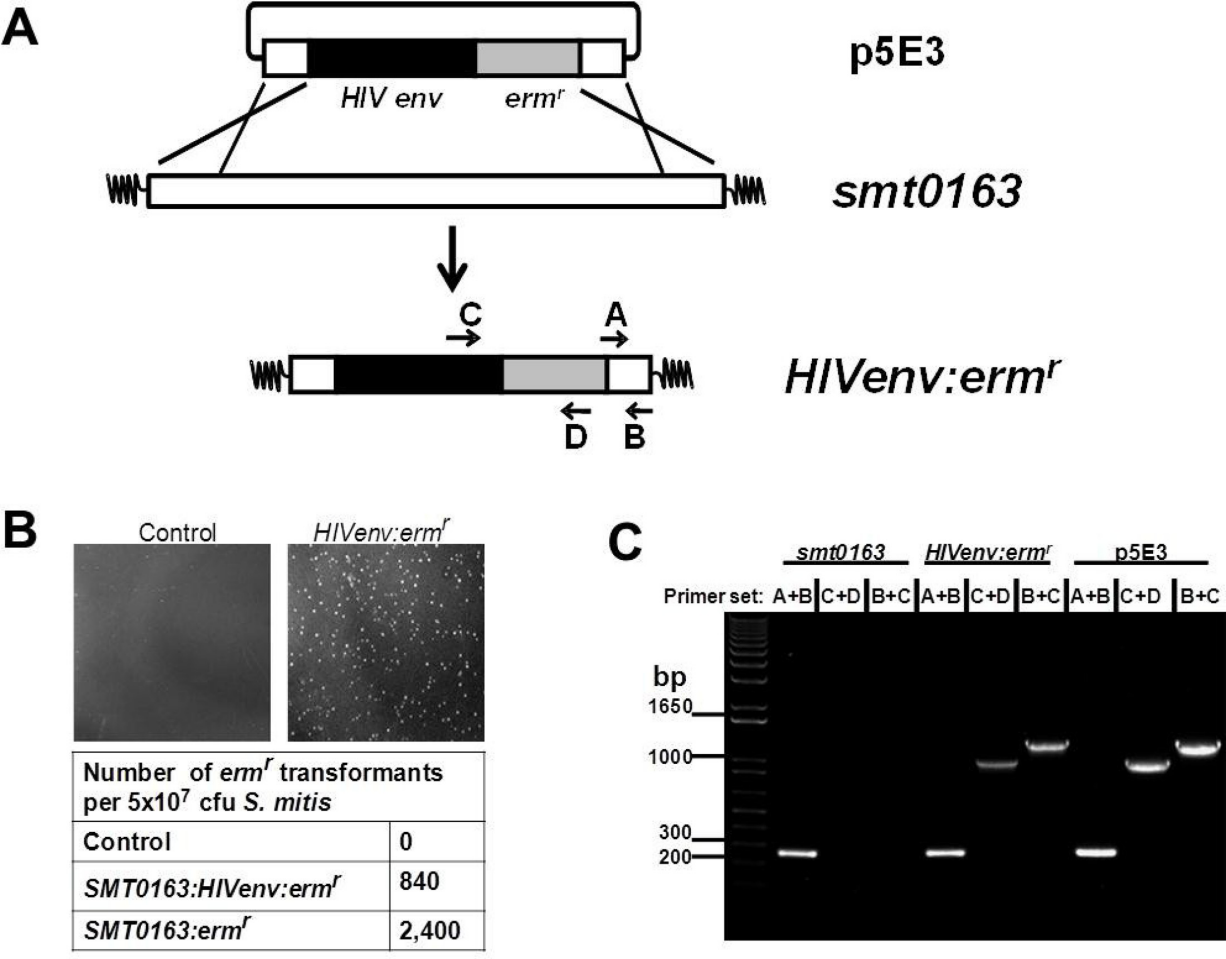
Construction of recombinant *S*. *mitis* expressing HIV Env gp120. (A) Recombinant *S*. *mitis* (*rS*. *mitis*) was generated by homologous recombination. The p5E3 suicide plasmid was created by inserting the *S*. *mitis* codon-optimized HIV-1 HXBc2 Env gp120-His-tag (HIV Env) in-frame with the 250bp 5’ end of the pullulanase gene (pulA/Smt0163) followed by ermr gene and the 250bp 3’ end of the pulA gene into pCR2.1 for transformation and integration into the Smt0163 locus. (B) *S*. *mitis* was transformed with water (control), p5E3 (*Smt0163*:*HIVenv*:*erm* ^*r*^) or pCR2.1 containing ermr flanked by 250bp pulA 5’ and 3’ fragments (*Smt0163*:*erm* ^*r*^). Growth of transformants on THB plates containing 50μg/ml erythromycin and transformation frequency are shown. (C) Integration was confirmed by PCR using *S*. *mitis*-specific primers A/B, HIV-specific primer C and ermr-specific primer D.

Recombinant *S*. *mitis* (*rS*. *mitis*) was generated by homologous recombination. The p5E3 suicide plasmid vector was created by inserting HIV-1 HXBc2 Env gp120-His-tag (HIV Env) [14] (codon-optimized for *S*. *mitis* expression by BlueHeron Technology, Bothel, WA) in-frame with the 250bp 5’ end of the pullulanase gene (pulA/Smt0163) followed by Erm^r^ gene [15] and the 250bp 3’ end of the pulA gene into pCR2.1 (Invitrogen, Carlsbad, CA) for transformation and integration into the Smt0163 locus (Fig 1A). *S*. *mitis* was transformed by electroporation with p5E3 to generate homologous recombinant *S*. *mitis* expressing HIV-1 Env or with pCR2.1 containing Erm^r^ flanked by 250bp pulA 5’ and 3’ fragments to generate control *S*. *mitis* empty vector. *rS*. *mitis* clones were selected on Todd Hewitt Broth (THB) plates containing 50 μg/ml erythromycin (Fisher Scientific, Pittsburgh, PA).

Expression of the HIV Env gp120 protein was assessed by Western blotting. Single colonies were grown in THB medium containing 50 μg/ml of erythromycin and grown 3–5 days until an optical density at 600 nm (OD600) approximately equal to 1. *rS*. *mitis* cells were then harvested and washed twice in ice-cold phosphate-buffered saline (PBS). Cell pellets were collected and bacterial lysates were generated by vortexing at top speed with 106 micron glass beads (Sigma, St. Louis, MO) for 15 minutes. Proteins from 10 ml culture supernatants were filtered through a 0.2-μm-pore-size filter to remove cell material and either concentrated 5x using a 10-kDa membrane filter (Amicon) or precipitated with either acetone or 10% trichloroacetic acid. Expression of the viral gp120 protein was assessed by Western blotting of bacterial lysates, concentrated or TCA- or Acetone-precipitated supernatants (1 μg of total protein) using Penta-His HRP (Qiagen, Hilden, Germany). Expression of HIV gp120 in concentrated supernatant was also assessed by binding with human patient sera recognizing HIV-1 gp120, which were pooled from HIV-infected individuals followed by a secondary goat anti-human IgG conjugated with HRP (ThermoFisher, Waltham, MA). For detection a chemiluminescence ECL kit was used according to the manufacturer's protocol (Invitrogen, Carlsbad, CA).

### Production of HIV-1 HXBc2 Env and *S*. *mitis* antigens

The HXBc2 Env gp120 gene [14] was subcloned into pET14b expression vector (Novagen-EMD Chemicals, Gibbstown, NJ), expressed in BL21(DE3) pLysS *E*. *coli* host (Invitrogen, Carlsbad, CA) and the over-expressed recombinant protein was purified by affinity chromatography under denaturing condition as we have previously described [16]. To produce *S*. *mitis* antigens, *S*. *mitis* was grown in THB media to OD value of 1.0. *S*. *mitis* cells were washed twice and resuspended in PBS. Bacterial lysate was generated by vortexing at top speed with 106 micron glass beads (Sigma, St. Louis, MO) for 15 minutes and filter sterilized.

### Mice, immunizations, blood and saliva collection

6–8 week old female Balb/c mice were purchased from Charles River Laboratories (Wilmington, MA) and kept under specific pathogen-free conditions at the Forsyth Institute Animal Facility. 6–8 week old germ-free Balb/c were housed at the Gnotobiotic and Microbiology Core (CHB) Facility, Brigham Women’s Hospital and Harvard Medical School, Boston, MA. All animal procedures were carried out under the guidelines of the Institutional Animal Care and Use Committee at the Forsyth Institute and the Animal Care and Use Committee at Harvard Medical School.

Mice were immunized with recombinant *S*. *mitis* grown in THB media containing 50 μg/ml erythromycin to OD600 value of 1.0. The viable vaccine vector was washed and resuspended in phosphate-buffered saline (PBS) and inoculated into mice orally at 10^9^ cfu or intraperitoneally at 2x10^8^ cfu in 200 μl PBS.

For saliva and blood collection, mice first received ketamine (100 mg/kg of body weight) with xylazine (12 mg/kg) anesthesia intraperitoneally. 100–200 μl blood was collected retro-orbitally from anesthetized mice using a sterile beveled tip. Saliva secretion was stimulated by subcutaneous injection of 0.1 ml 0.1mg/ml carbachol and approximately 100–200 μl saliva was collected from each animal.

### Colonization study

*rS*. *mitis* colonization of mice was determined by the presence of the oral bacterium in the mouth and feces. The upper right buccal cheek of each mouse was scrubbed with calcium alginate oral swab (Fisher Scientific, Pittsburgh, PA), which was then immersed in 1 ml THB media and vortexed thoroughly. Two fecal pellets per mouse were collected and resuspended vigorously in 1 ml THB media by vortexing. Undiluted and diluted oral and fecal samples were spread on THB agar plates, containing 50 μg/ml erythromycin to determine the total number of colony-forming-units (cfu) present in the collected feces. The cfu counts were normalized against the wet weight of the fecal pellets.

### Measurement of Antibody Responses

High-binding 96-well microplates (Costar, Lowell, MA) were coated with the purified HIV-1 HXBc2 Env protein (2 μg/ml) or *S*. *mitis* lysate antigens (2 μg/ml) prepared in 0.2 M sodium carbonate/bicarbonate buffer (pH 9.6) and incubated overnight at 4°C. Wells were washed with PBS containing 0.05% Tween-20 (PBS-T) and blocked with 1% BSA in PBS-T for 2 hr. Serum or saliva samples were diluted 1:3 and added at 2- to 3-fold serial dilutions in PBS-T containing 0.1% BSA and plates were incubated for 1 hr at room temperature. After another washing step, horseradish peroxidase, HRP-conjugated rat anti-mouse IgA (clone 11-44-2), IgG1 (clone SB77e) or IgG2a (clone SB84a) (Southern Biotech, Birmingham, AL) was added at 1:4000, 1:5000, 1:5000 dilution, respectively and incubated for 1 hr. A substrate solution containing tetramethylbenzidine (KPL, Gaithersburg, MD) was added and colour development was stopped using 1 m HCl. Optical density data were recorded as absorbance at 450 nm. OD values twice above the background were considered positive and values between 0.01 and 1.0 were used to determine the amount of antibody produced and adjusted according to the dilution factor to determine the OD value of the undiluted saliva and serum samples.

### Intracellular cytokine staining

Blood collected from anesthetized mice was mixed with PBS containing 10mM EDTA to prevent coagulation and peripheral blood mononuclear cells (PBMCs) were purified using lympholyte-M (Cedarlane, Burlington, NC). 1–2 x 10^5^ PBMCs were cultured at 37°C in a 5% CO2 environment for 6 hr in the presence of RPMI-1640/10% fetal calf serum alone or with either 10 μg/ml *S*. *mitis* lysate or 10 μg/ml HIV HXBc2 envelope protein. All cultures contained Monensin (GolgiStop; BD Biosciences, San Jose, CA). The cultured cells were cell-surface stained with the following monoclonal antibodies purchased from BD Biosciences: anti-CD3-FITC (145-2C11), anti-CD4-allophycocyanin-Cychrome7 (GK1.5), anti-CD8α-perdinin chlorophyll protein-Cychrome 5·5 (53–6.7). After fixing with Cytofix/Cytoperm solution (BD Biosciences), cells were permeabilized and stained with anti-IFNγ/allophycocyanin (XMG1.2), anti-TNFα/phycoerythrin-Cychrome 7 (MP6-XT22), and anti-interleukin-2 (IL-2) -phycoerythrin (JES6-5H4). Labelled cells were fixed in 1% formaldehyde-PBS. Samples were collected on a BD FACS Aria III flow cytometer (BD Biosciences) and analyzed using flowjo software (Tree Star, Ashland, OR). Approximately 50–100,000 events were collected per sample. Doublets were excluded by forward scatter-area versus forward scatter-height. The CD4+ and CD8+ T cells were determined by their expression of CD3, CD4 or CD8 and the frequency of cells producing IFNγ, TNFα and IL-2 was determined using flowjo (Tree Star). All values used for analysis are background subtracted. Responses were considered positive when the percentage of total cytokine-producing cells was at least twice that of the background.

### Multiplex Luminex Assay

Cytokines present in culture supernatants of stimulated cells were quantitated using MILLIPLEX^®^ MAPmultiplex cytokine biomarker magnetic bead panel for detection of mouse IL-2, IL-4, IL-6, IL-10, IL-13, IL-17A, TGFβ, TNFα and IFNγ (EMD Millipore, Darmstadt, Germany). Samples were analyzed in a microplate well using Luminex^®^ 200^™^ (Luminex, Austin, TX).

### Statistical analysis

Data were expressed as means ± standard error of the means (SEM). Statistical tests were performed using Student’s *t* test. A *P* value of less than 0.05 was considered significant.

## Results

### Construction of the prototype recombinant *S*. *mitis* HIV vaccine vector

To generate recombinant *S*. *mitis (rS*. *mitis)*, strain NCTC 12261 was transformed with DNA fragment p5E3 containing the HIV Env gene to allow homologous recombination and integration into the pullulanase (*pulA*/Smt0163) gene (Fig 1A). The *pulA*/Smt0163) gene was selected because it is non-essential and highly expressed [17]. The p5E3 DNA fragment consists of the pCR2.1 plasmid containing the *S*. *mitis* codon-optimized HIV-1 HXBc2 gp120 *env*-His-tag (HIV Env) in-frame with the 250bp 5’ end of the pullulanase gene (*pulA*/Smt0163), followed by an Ermr gene and the 250bp 3’ end of the Smt0163 gene (Fig 1A). Transformation frequencies on erythromycin-containing plates were 840 and 2,400 transformants per 5 x10^7^ cfu bacteria added for plasmids containing the HIVenv:erm^r^ or erm^r^ gene only, respectively (Fig 1B). Integration of the HIV gene was confirmed by PCR and sequencing. PCR analysis showed that the *S*. *mitis*-specific primers A/B generated the expected 250 bp *smt0163* PCR product, while the HIV-specific primer C and *erm* ^*r*^-specific primer D generated the expected 1 kb product (Fig 1C). Sequencing of the PCR products further showed the integration of the HIV Env gene in *S*. *mitis* (data not shown). Thus, we successfully constructed *rS*. *mitis* containing an HIV gene integrated at the *pulA* gene locus of the *S*. *mitis* genome.

### Recombinant *S*. *mitis* expresses HIV-1 envelope protein

**Fig 2:**
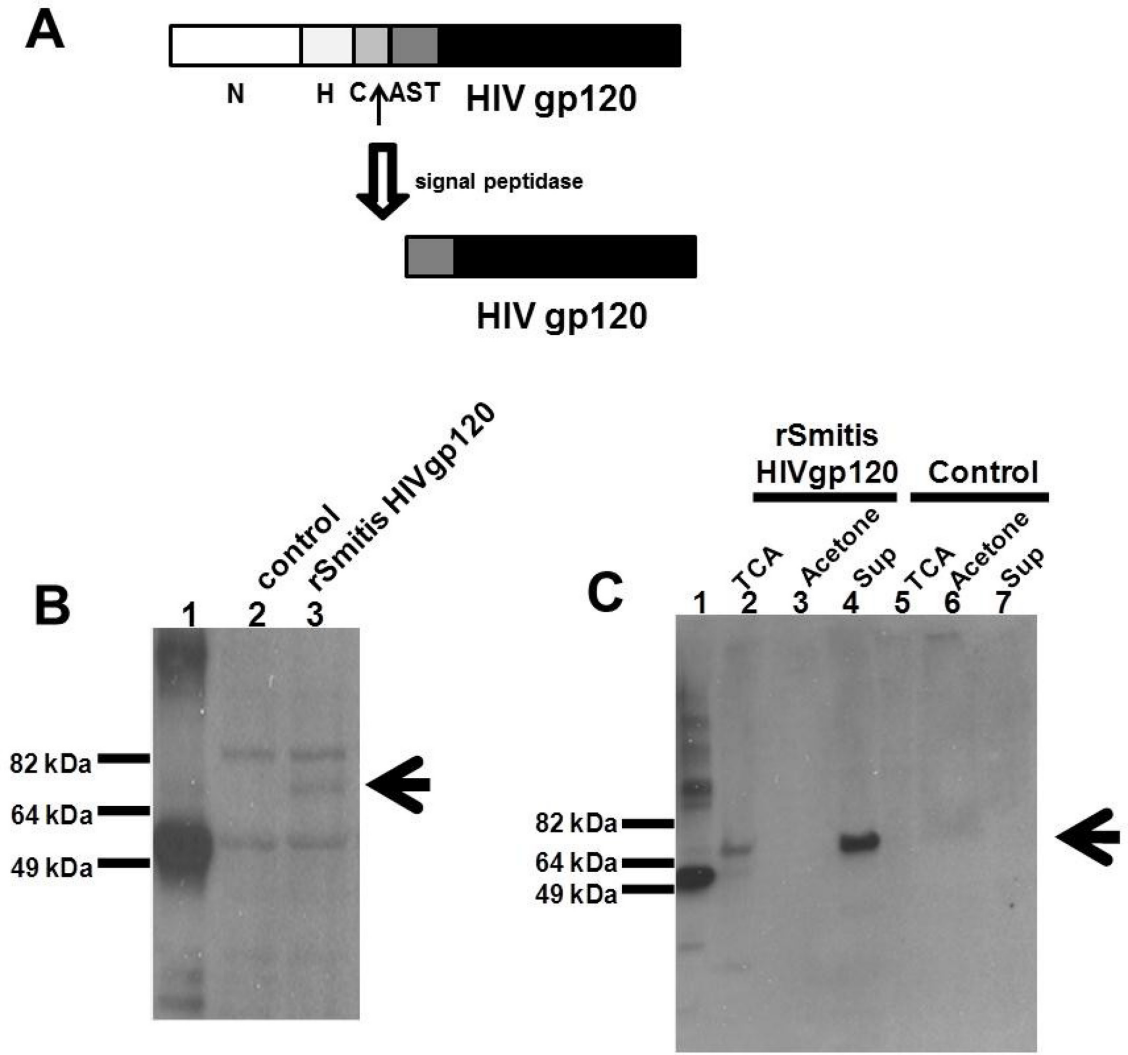
Recombinant *S*. *mitis* expresses HIV envelope protein. *rS*. *mitis* with the integrated HIV HXBc2 Env gp120 was designed to secrete HIV Env by ligating the HIV Env in frame with 250bp 5’ end of the pullulanase gene (pulA/Smt0163) encoding a signal peptide that allows processing and secretion of the HIV antigen. (A) The signal peptide has an amino-terminal region (N), a hydrophobic core (H), a signal peptidase cleavage site (C), and an accessory Sec transport motif (AST). Expression of HIV Env containing a C-terminal His tag was assessed by Western blotting using Penta-His-HRP from a representative recombinant clone in *S*. *mitis* lysates (B) and in culture supernatants (C) by TCA-precipitation (TCA), acetone precipitation (Acetone) and Amicon filter-concentration (Sup). HIV Env expression in lysates (B) and supernatants of control *S*. *mitis* vector (control) (C) is shown. The arrow denotes expression of the Env Ag band. 100 ng of His-tagged *M*. *tuberculosis* protein (MT0401) was used as a positive control (B and C, lane 1). (D) The expression of HIV-1 gp120 in *rS*. *mitis* containing the HIV Env gene (lane 2) in Amicon filter-concentrated supernatant was detected using human HIV patient sera. *rS*. *mitis* containing the empty plasmid was used as a negative control (lane 1).

Our strategy was to express foreign vaccine antigens in the secreted form, which enhances the immunogenicity of bacterial antigens [14]. The constructed *rS*. *mitis* contains the integrated *S*. *mitis* codon-optimized HIV-1 HXBc2 *env*-His-tag (HIV Env) in-frame with the 250bp 5’ end of the pullulanase gene (*pulA*/Smt0163) encoding a signal peptide that allows processing and secretion of the HIV antigen (Figs 1A and 2A) [18, 19]. The signal peptide has a predicted amino-terminal region (N), a hydrophobic core (H), a signal peptidase cleavage site (C), and an accessory Sec transport motif (AST).

Next, we determined the expression of HIV Env gp120 antigen containing the C-terminal His tag in *S*. *mitis*. HIV Env gp120 in the bacterial pellets and as a secreted product in the supernatants was detected by Western blotting using anti-His tag antibody (Penta-His-HRP). HIV Env gp120 linked to the PulA signal peptide was expressed in *S*. *mitis* lysates of a representative recombinant clone (Fig 2B, lane 3). Lysates also contained proteins that cross-react with the anti-His antibody. In addition, secreted Env gp120 was detected in bacterial culture supernatants (Fig 2C, lane 4). 100 ng of His-tagged *M*. *tuberculosis* protein (MT0401) was used as a positive control [20] (Fig 2B and 2C, lane 1). The Env Ag was present in the TCA-precipitated (Fig 2C, lane 2) and Amicon filter-concentrated supernatant (Fig 2C, lane 4), although not in acetone precipitates (Fig 2C, lane 3). As expected, the HIV Env was not expressed in lysates (Fig 2B, lane 2) and supernatants of the control *S*. *mitis* empty vector (Fig 2C, lanes 5–7). Additionally, we determined the expression of HIV-1 gp120 in the concentrated supernatant using human HIV patient sera and found that the *rS*. *mitis* containing the Env gp120 but not control *rS*. *mitis* expresses HIV g120 (Fig 2D). The apparent molecular weight of gp120 was similar to the expected molecular weight (of approximately 70 kDa), suggesting that Env likely was not glycosylated in *S*. *mitis*.

### Expression of HIV Env in *rS*. *mitis* is stable

**Fig 3:**
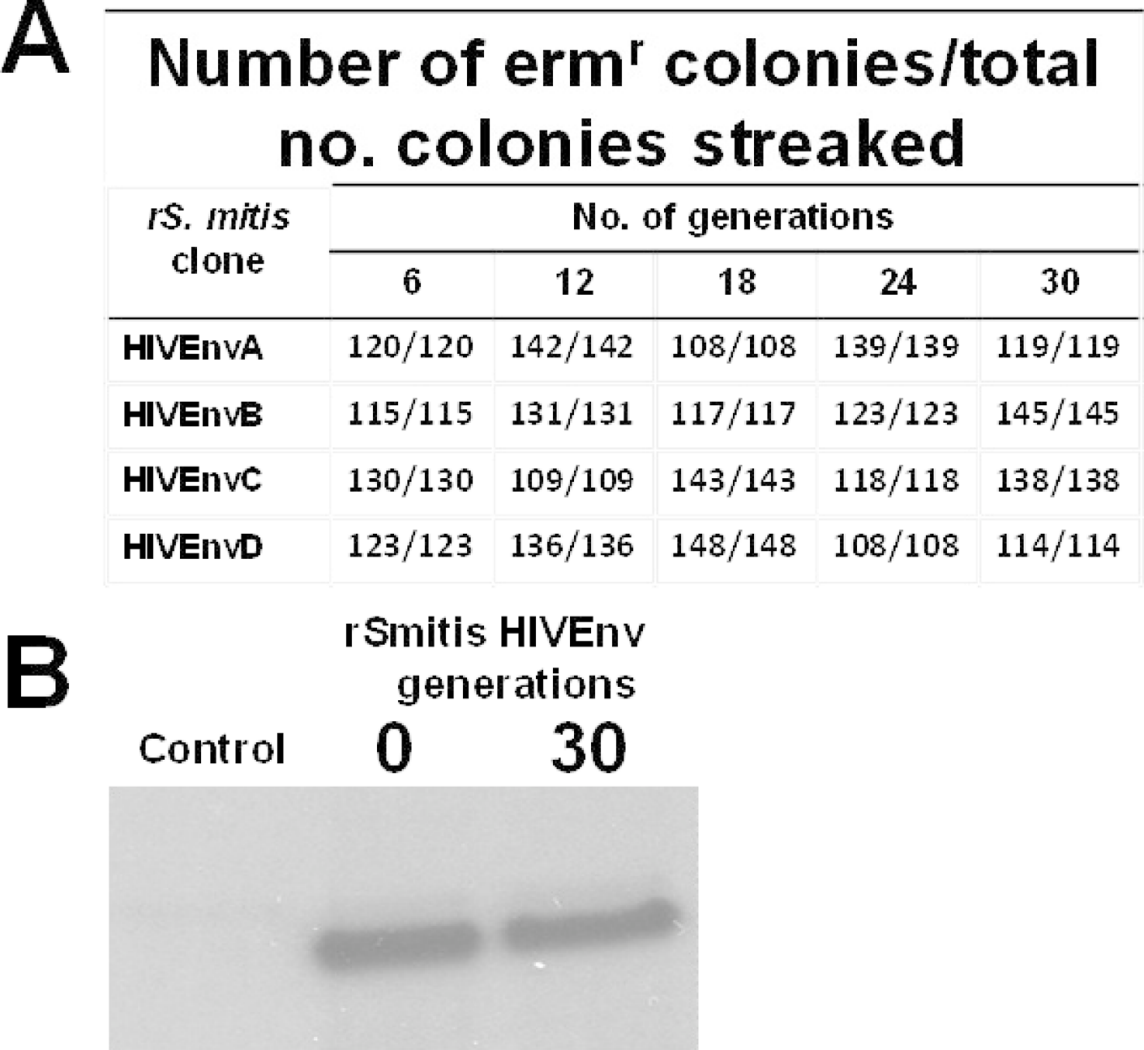
*rS*. *mitis* expressing HIV Env is stable. To determine the stability of the integrated Env gp120 gene in *rS*. *mitis*, four clones (HIVEnvA, B, C, and D) were picked at random and grown anaerobically for 24 hours which represents approximately three generations (7.1 hours per generation) in THB media without erythromycin. Cultures were grown for approximately 30 generations without erythromycin. From each generation 100 and 150 colonies were picked at random and streaked to THB plates with and without erythromycin. For each *S*. *mitis* HIV Env clone, the number of Erm^r^ colonies/total number of colonies streaked after 6, 12, 18, 24, and 30 generations was determined (Fig 3A). Expression of Env in the same daughter clones was analyzed by Western blot analysis. Env production in a representative daughter clone (after 30 generations) and the original HIVgp120A clone is shown (Fig 3B).

To determine the stability of the integrated HIV-1 Env gp120 gene in *rS*. *mitis*, four clones were picked at random and grown anaerobically for 30 generations. Loss of erythromycin-resistance was taken as an indication of the loss of the Env transgene. The progeny from all four clones were stable, with no loss of erythromycin resistance after ten subcultures or 30 generations of replication without antibiotic (Fig 3A). In addition, we analyzed the expression of Env in the same daughter clones. Western blot analysis revealed that they secreted the same amount of Env antigen as the original *rS*. *mitis* clones; Env production in a representative daughter clone after 30 generations and the original HIVgp120A clone is shown (Fig 3B). These results indicate that our *rS*. *mitis* is stable, a vaccine property that makes it a good candidate for further preclinical testing.

### Recombinant *S*. *mitis* vaccine vector abundantly and persistently colonized germ-free mice but not conventional mice

**Fig 4:**
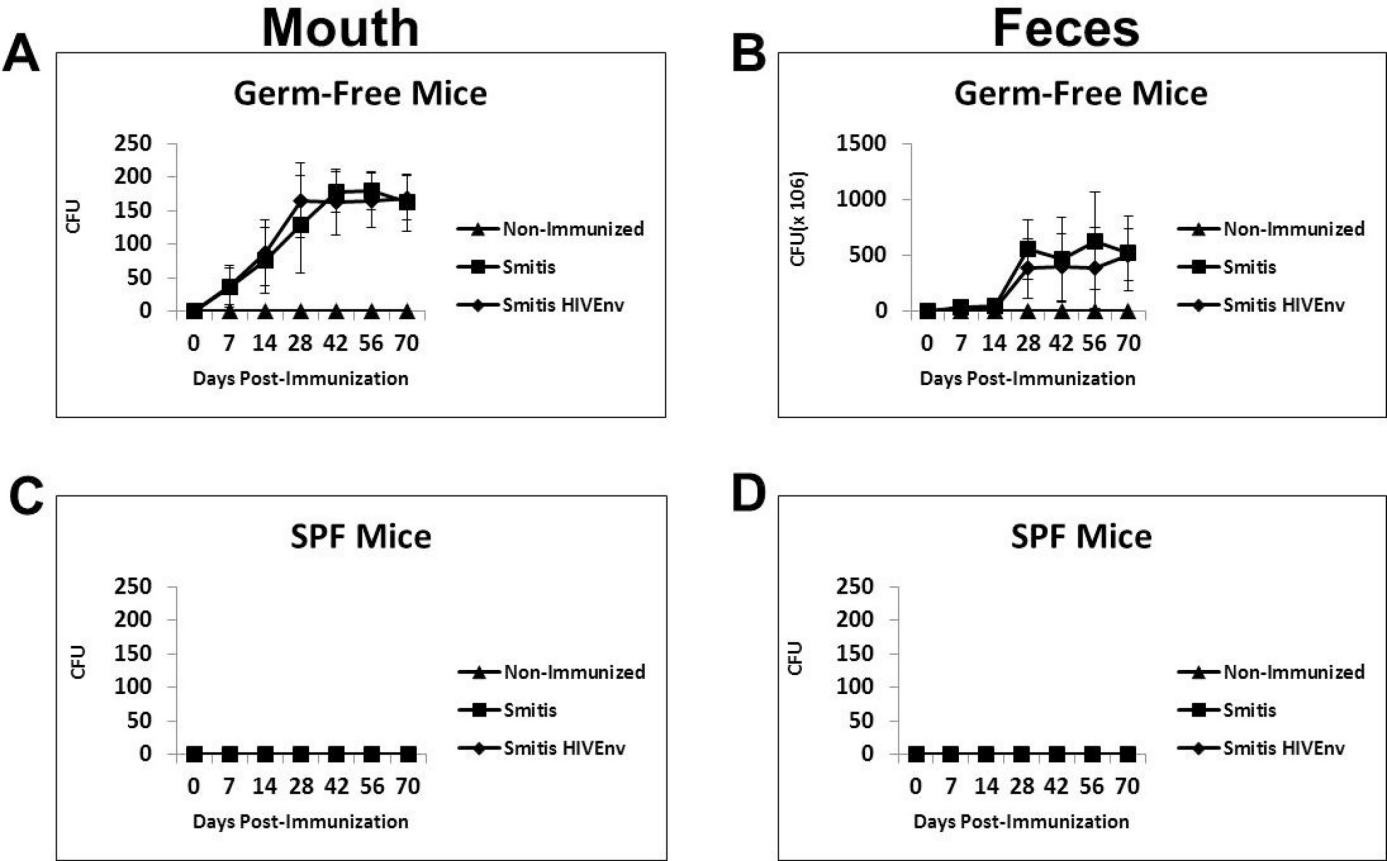
Recombinant *S*. *mitis* colonizes germ-free mice efficiently and persistently. Germ-free Balb/c mice and conventional SPF mice were inoculated orally with 10^9^ cfu *rS*. *mitis* expressing HIV Env gp120 (Smitis HIV Env), *rS*. *mitis* containing an integrated Erm^r^ gene without Env (Smitis) or PBS (Non-Immunized). Following inoculation the upper right buccal cheek was swabbed and two pellets of feces were collected from each mouse at various time points. *rS*.*mitis* colonization was assessed by growth on THB plates containing 50 μg/ml erythromycin. The mean colony-forming-units, cfu (±SEM) from 3 mice/group at various timepoints, present in the mouth (A) and feces (B) of germ-free mice and in the mouth (C) and feces (D) of conventional mice inoculated with *rS*. *mitis* are shown.

Oral pioneer commensal bacteria such as *S*. *mitis*, *S*. *salivarius* and *S*. *oralis* are excellent colonizers of the mouth. Therefore, a suitable animal model to test the preclinical efficacy of recombinant *S*. *mitis* must be able to be effectively colonized. A prior study showed that gnotobiotic or germ-free mice were colonized by human oral bacteria, including *S*. *mitis* [21]. To assess whether germ-free mice are a suitable animal model, germ-free mice as well as conventional mice were inoculated orally with 10^9^ cfu *rS*. *mitis* expressing HIV Env gp120 (Smitis HIVEnv) and control *S*. *mitis* (Smitis). Following oral vaccination, colonization of the mouth was observed at day 7 and the number of bacteria continued to increase over time, peaking on day 30 and remained persistently high thereafter (Fig 4A). Both *rS*. *mitis* HIV Env and control *S*. *mitis* colony-forming-units were significantly higher in immunized compared to non-immunized mice on day 7, 14, 28, 42, 56 and 70 (p < 0.05 at each timepoint). On those same days, *rS*. *mitis* was also found abundantly in the feces of the immunized mice but not in non-immunized mice (Fig 4B). Conventional SPF mice were not colonized by *rS*. *mitis* (Fig 4C and 4D). These results demonstrate that germ-free mice are efficiently and persistently colonized by *S*. *mitis* and therefore are a viable animal model for the preclinical testing of *rS*. *mitis* vaccine vectors.

### Recombinant *S*. *mitis* vaccine vector induces antigen-specific salivary and systemic antibody responses in colonized mice

**Fig 5:**
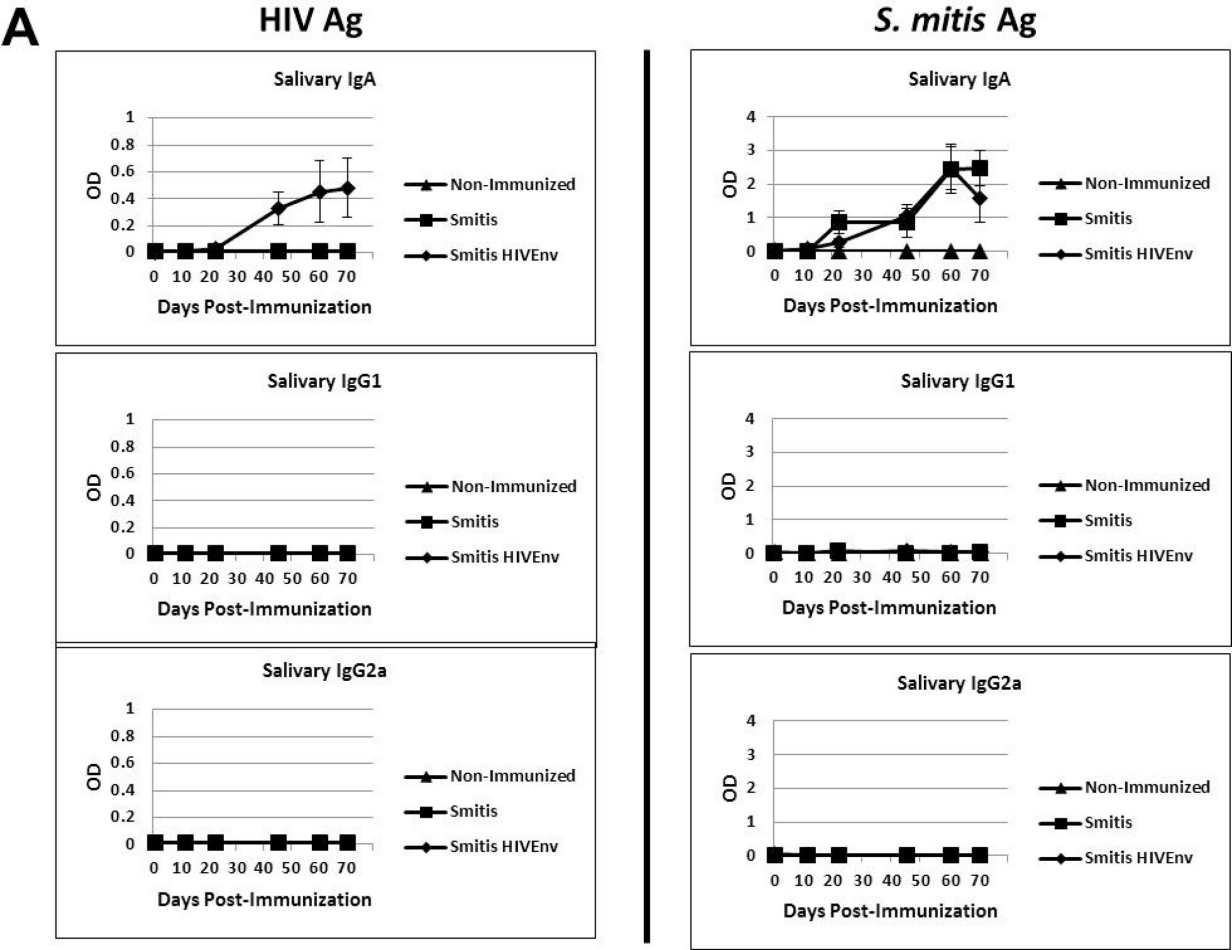
Recombinant *S*. *mitis* induces mucosal and systemic antibody responses. Germ-free Balb/c mice were inoculated orally with 10^9^ cfu *rS*. *mitis* expressing HIV Env gp120 (Smitis HIV Env), *rS*. *mitis* containing an integrated Erm^r^ gene without Env gp120 (Smitis), or PBS (Non-Immunized). Following immunization, the presence of IgA, IgG1, and IgG2a antibodies specific to the HIV Env gp120 protein (HIV Ag) or *S*. *mitis* lysate antigens (*S*. *mitis* Ag) was measured by ELISA in the saliva (A) and serum (B) of mice. The mean optical density (OD) (±SEM) values of the undiluted samples from 3 mice/group at various timepoints post-immunization are shown. The saliva and serum antibody dilution factor was 1:3 or indicated otherwise.

Infants, children and adults colonized by *S*. *mitis* develop salivary antibodies to the pioneer bacterium [7, 22], and therefore we determined whether the vaccinated germ-free mice also develop antibody responses to *rS*. *mitis*. Salivary IgA specific to HIV Env protein as well as *S*. *mitis* lysate antigens were initially detected three weeks post-inoculation and the antibody responses increased concomitant with *S*. *mitis* persistence in the oral cavity (Figs 4A and 5A). The amount of HIV-specific salivary IgA in *rS*. *mitis* HIV Env-vaccinated mice was significantly higher than in control *S*. *mitis*-vaccinated or in non-immunized mice on day 22, 45, 60 and 70 (p < 0.05 at each timepoint). As expected, on those same days, similar amounts of *S*. *mitis* antigen-specific salivary IgA were found in either *rS*. *mitis* HIV Env or control *S*. *mitis*-immunized mice, which were both significantly higher than in non-immunized mice (p < 0.05 at each timepoint). Salivary IgG1 and IgG2a antibodies specific to *rS*. *mitis* were undetectable.

Systemic antibody responses were also generated in the vaccinated mice. The *rS*. *mitis* vaccine-elicited serum IgA, IgG1 and IgG2a specific to HIV in *rS*. *mitis* HIV Env colonized but not in mice colonized by control *S*. *mitis* (Fig 5B). The amount of HIV-specific serum IgA and IgG1 were significantly higher in *rS*. *mitis* HIV Env-vaccinated mice than in control *S*. *mitis*-vaccinated or in non-immunized mice on day 16, 31, and 58 (p < 0.05 at each timepoint), while the HIV-specific IgG2a concentrations were significantly higher on day 31 and 51 in mice immunized with *rS*. *mitis* HIV Env than in mice immunized with control *S*. *mitis* and non-immunized mice (p < 0.05 at each timepoint). As expected, serum IgA, IgG1 and IgG2a responses specific to *S*. *mitis* lysate antigens were found in both *S*. *mitis*-vaccinated groups (Fig 5B). Compared to the levels of antibodies found in non-immunized mice, the amount of *S*. *mitis* antigen-specific serum IgA and IgG1 in either *S*. *mitis*-vaccinated group were significantly higher on days 16, 31, and 56, while the antigen-specific serum IgG2a were significantly higher on day 31 and 56 (p < 0.05 at each timepoint). Taken together, these results show that *rS*. *mitis* is capable of eliciting robust mucosal and systemic antibody responses in vaccinated animals.

### Recombinant *S*. *mitis* vaccination induces systemic T cell non-responsiveness, likely due to oral tolerance

**Fig 6:**
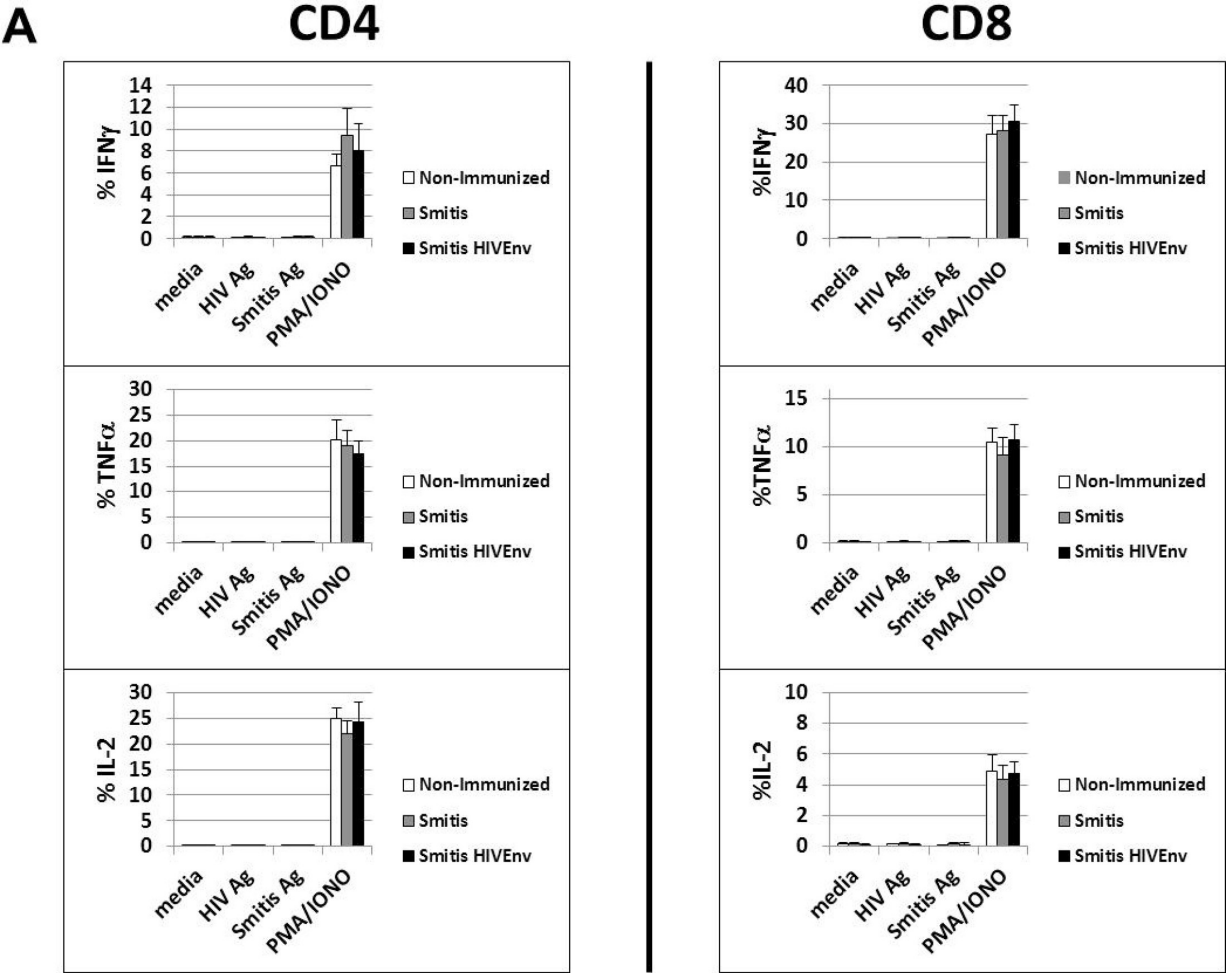
*rS*. *mitis* induces systemic T cell tolerance. (A) Germ-free mice were vaccinated orally with 10^9^ cfu *rS*. *mitis* expressing HIV Env gp120 (Smitis HIV Env) or *rS*. *mitis* containing an integrated Erm^r^ gene without Env gp120 (Smitis). Ten weeks later, peripheral blood monuclear cells were isolated from vaccinated mice and stimulated with media alone (media), *S*. *mitis* lysate antigens (*S*. *mitis* Ag), the HIV Env gp120 protein (HIV Ag), or PMA and Ionomycin (PMA/IONO). Production of IFNγ, TNFα, and IL-2 by the stimulated cells measured by % IFNγ+, TNFα+, and IL-2+ CD4 or CD8 T cells from each group (3 mice/group) is shown. (B) To assess tolerance induction, germ-free mice were inoculated orally with 10^9^ cfu to allow colonization and three months later, these mice were injected intraperitoneally (IP) with 2x10^8^ cfu r*S*. *mitis* HIV Env (Balb/c (colonized) IP group). Non-colonized mice were either non-immunized (Non-immunized group) or inoculated IP with 2x10^8^ cfu r*S*. *mitis* HIV Env (Balb/c IP group). Two weeks after intraperitoneal immunization of the colonized and non-colonized mice, the spleen and mesenteric lymph nodes (MLN) were harvested and stimulated in vitro with media, 10μg/ml purified HIV Env gp120 protein (HIV Ag), 10μg/ml of *S*. *mitis* antigens (Smitis Ag), Concanavalin A (ConA), or PMA/IONO. T cell proliferative responses were measured by using 3H-Thymidine incorporation assay. Mean CPM from 3 mice/group is shown. The production of IL-2, IL-4, IL-6, IL-10, IL-13, IL-17A, TGFβ, TNFα and IFNγ by cells stimulated with media, HIV antigen, or *S*. *mitis* antigens was measured using multiplex luminex assay (C). The assay was performed in duplicates and the mean concentration (pg/ml) for each cytokine from pooled supernatants samples (3 mice/group) is shown.

The nature of the T cell response to *S*. *mitis* in humans is not clearly known. We investigated whether *rS*. *mitis* induced T cell responses in the vaccinated germ-free mice. Vaccinated mice that harbored *rS*. *mitis* persistently for three months developed no vaccine-specific T cell-responses systemically. Intracellular cytokine staining analysis revealed that peripheral blood T cells isolated from vaccinated mice stimulated with either HIV or *S*. *mitis* antigens did not produce IFNγ, TNFα and IL-2 (Fig 6A). In contrast, T cells expressed large amounts of IFNγ, TNFα and IL-2 in response to PMA and Ionomycin stimulation (Fig 6A).

We explored the possibility that the observed T cell non-responsiveness in *rS*. *mitis*-vaccinated mice is due to the induction of oral tolerance. Previous studies of oral T cell tolerance show that systemic T cells from orally tolerized mice do not respond when challenged with antigen in vivo [23]. Germ-free Balb/c mice were either previously orally vaccinated (colonized) or non-vaccinated and three months later, they were injected intraperitoneally (IP) with 2x10^8^ cfu *rS*. *mitis* HIV Env. Two weeks after IP injection, splenocytes and mesenteric lymph node lymphocytes were isolated and proliferative and cytokine responses to HIV and *S*. *mitis* lysate antigens were assessed. In mice previously vaccinated and colonized with *rS*. *mitis* followed by systemic IP injection of *rS*. *mitis* three months later (Balb/c (colonized) IP group), in vitro proliferative and cytokine responses in the spleen and MLN to HIV and *S*. *mitis* antigens were not seen (Fig 6B and 6C). In contrast, lymphocytes isolated from non-orally colonized germ-free mice that received IP injection with *rS*. *mitis* (Balb/c IP group) showed significant proliferative responses (p < 0.05 for spleen and MLN) to HIV and *S*. *mitis* antigens compared to media only control as well as to lymphocytes isolated from those mice that were previously colonized by *rS*. *mitis* (Balb/c (colonized) IP group). In addition, the lymphocytes from the *rS*. *mitis* IP-injected mice produced various cytokines in response to in vitro stimulation with HIV and *S*. *mitis* antigens. Using multiplex Luminex assay, the stimulated splenocytes and MLN cells were found to produce IL-2, IL-4, IL-6, IL-10, IL-13, IL-17, TNFα and IFNγ (Fig 6C). As expected, spleen and MLN cells from mice that were neither orally vaccinated nor IP injected with *rS*. *mitis* (Non-immunized group) did not proliferate and produced little or no cytokines (Fig 6B and 6C). Altogether, these results suggest that oral immunization and persistent colonization by *rS*. *mitis* induces systemic T cell non-responsiveness likely due to oral T cell tolerance.

## Discussion

*Streptococcus mitis* has unique biologic features that make this oral bacterium an attractive vaccine vector against HIV/AIDS. *S*. *mitis* is a commensal nonpathogenic bacterium in pediatric and adult populations and therefore should be safe clinically. The sequence of the *S*. *mitis* genome is available and the organism is genetically tractable for expression of HIV immunogens. *S*. *mitis* is most abundant in the mouth and persistently colonizes oral surfaces and the nasopharynx of infants and adults, and this microbe has been shown to induce durable mucosal immunity. We constructed recombinant *Streptococcus mitis* stably expressing the model HIV HXBc2 envelope antigen and performed preclinical immunogenicity studies in mice to evaluate this delivery system as a possible mucosal AIDS vaccine. Germ-free mice were found to be a good animal model for the preclinical evaluation of *rS*. *mitis*. Germ-free mice supported the growth and colonization of the vaccine vector. *rS*. *mitis* efficiently and persistently colonized the mouth of germ-free mice, similar to what is seen in humans. Conventional SPF mice, on the other hand, were not colonized by *rS*. *mitis*, perhaps due to the inability of *S*. *mitis* to compete against resident microbes, or due to the presence of cross-reactive mucosal immune responses elicited by resident microbes that prevents *S*. *mitis* colonization.

Vaccine immunogenicity studies in the germ-free mouse model reveal the ability of *rS*. *mitis* to elicit preferentially antigen-specific mucosal and systemic immune responses. Salivary IgA responses were generated three weeks after vaccination. Serum IgA and IgG1 were detected two weeks after vaccination while IgG2a responses were seen approximately 4 weeks after vaccination. Antibody responses specific to the HIV Env vaccine and vector antigens subsequently increased over time as *rS*. *mitis* persisted in the host. Consistent with these findings, we previously found that *rS*. *mitis* expressing the Mtb 85b also induced systemic and mucosal antibodies in gnotobiotic pigs which were also colonized by the oral vaccine vector [24]. Conventional SPF mice, despite not capable of being colonized, also developed HIV-specific antibody responses to *rS*. *mitis* but only after repeated immunizations (data in S1 Fig). Collectively, these findings suggest that persistence and continuous exposure to antigen is necessary for the generation of mucosal and systemic antibody responses to *rS*. *mitis*.

Since HIV-1 Env is not glycosylated in *S*. *mitis*, expression of neutralizing Env gp120/gp41 epitopes independent of glycosylation will be necessary. Another viable approach is to prime with *rS*. *mitis* followed by boost with relevant Env immunogens may be needed to elicit broadly neutralizing antibodies. A possible boosting Env immunogen may include improved glycosylated HIV Env gp120/gp41 trimer constructs that will elicit broadly neutralizing antibodies [25]. These vaccine approaches will be explored and antibodies generated will be assessed for their ability to neutralize HIV. Mucosal antibody responses in the gut and vagina of *rS*. *mitis*-vaccinated germ-free mice are currently being assessed, since these mucosal surfaces are likely portals of HIV entry.

Our findings suggest that mucosal and systemic antibody responses are elicited a few weeks after vaccination and that propensity of *S*. *mitis* vaccine vectors to elicit mucosal and systemic antibody responses consistent with what was observed in humans is an attractive vaccine feature that may make *rS*. *mitis* effective at preventing HIV infection via mother-to-child and adult-to-adult transmission. Cross-sectional and longitudinal analyses of infants from birth to 2 years of age show that within the first month of birth sIgA antibodies recognizing *S*. *mitis* can readily be detected and IgA responses continue to persist over the 2–3 year observation [26]. The ability of oral *S*. *mitis* to elicit mucosal immunity early (within the first month after birth) may help prevent breast milk transmission of HIV because babies are estimated to become infected between 2 and 28 weeks of age [27]. With no intervention, the estimated cumulative risk of infant HIV-1 infection or death between 2 and 28 weeks is 7.0%. In light of these studies, an *S*. *mitis* vaccine vector can be developed to induce anti-HIV mucosal immunity soon after birth that will protect infants from contracting HIV from their infected mothers via breastfeeding. How soon after birth anti-HIV antibodies actually develop after *rS*. *mitis* vaccination in humans will be critical in that it may be necessary to treat infants with anti-retroviral drugs in the first few weeks to prevent HIV infection until protective antibodies against HIV are generated.

T cell responses to *S*. *mitis* in humans have not been fully investigated. Our immunogenicity study showed that vaccinated mice, despite being continuously exposed to a high dose of *S*. *mitis* antigens as a result of being efficiently and persistently colonized by *rS*. *mitis*, failed to develop systemic T cell responses. We began to explore the possibility that the systemic T cell non-responsiveness seen in *S*. *mitis* colonized germ-free mice could likely be due to oral tolerance. Oral tolerance studies show that systemic T cells from mice tolerized orally fail to respond to antigenic challenge in vivo [23]. Indeed, T cells from germ-free mice previously oral vaccinated and colonized with *rS*. *mitis* did not respond to intraperitoneal injection with *rS*. *mitis*. In contrast, germ-free mice injected systemically with *rS*. *mitis* generated HIV and *S*. *mitis* antigen-specific T cell responses. These results suggest that *rS*. *mitis* vaccination and persistent colonization induces systemic T cell tolerance. Human T cell clones specific to *S*. *mitis* antigens have previously been isolated in culture suggesting that systemic T cells are not tolerant to *S*. *mitis* antigens. However, stimulation with mitogen and IL-2 for the expansion of the T cell clones from highly purified memory T cells could have led to the reversal of tolerant T helper cells induced naturally by *S*. *mitis* resident in the human oral cavity [8].

Persistent and efficient colonization by *S*. *mitis* likely leads to high dose and persistent stimulation of the oral mucosa as well as the gut immune system since a large number of *rS*. *mitis* bacteria was found both in the mouth and in the feces. As a result, systemic T cell tolerance is induced concomitant with the generation of strong mucosal and systemic antibody responses to *rS*. *mitis*. Similar to systemic tolerance induced through oral administration of large dose of antigen, *rS*. *mitis* oral colonization could induce early T cell activation and TCR modulation, followed by an intermediate stage of anergy and subsequent deletion of *rS*. *mitis*-specific T cells after prolonged and persistent colonization by the oral bacterium [28]. If systemic T cells were tolerant, then why were there systemic antibody responses in the vaccinated animals? One likely explanation is that during the initial period of T cell activation prior to tolerance induction, T helper cells likely stimulated antigen-specific B cell responses that were seen in *rS*. *mitis* colonized mice.

The fact that r*S*. *mitis* induces systemic T cell tolerance may or may not affect the efficacy of the vaccine against HIV. We argue that an effective AIDS vaccine could elicit a robust mucosal antibody response that will prevent the penetration of the gut, oral, vaginal tissues which are the portals of HIV entry. Therefore, the ability of *S*. *mitis* to induce mucosal antibody responses could potentially inhibit HIV penetration of the mucosa. If mucosal antibody response were able to prevent penetration of the mucosa it might not be an issue whether the *S*. *mitis* induces systemic T cell tolerance for the virus would not be able to infect the gut and disseminate systemically. The ability of *S*. *mitis* to induce systemic antibody responses may also be effective if the HIV virus were able to penetrate the mucosa despite the presence of anti-HIV mucosal antibodies. To date, T cell-based vaccines against HIV have been tested in the Merck trial and HVTN human trials and both have failed. We need to explore antibody-based vaccines such as r*S*. *mitis*. Indeed, the Thai human trial (RV144) showed that antibody responses may play a role in protection against HIV [29]. Taken together, the possible efficacy of r*S*. *mitis*-based vaccines needs to be explored further.

Ongoing studies include delineating the mechanism(s) involved in the *rS*. *mitis*-induced T cell tolerance, which may involve suppression by T regulatory cells, T cell exhaustion, anergy, deletion, or IgA-mediated suppression [30–36]. In addition, the kinetics of persistence relative to development of systemic T cell tolerance is also being investigated to develop a vaccine strategy that would either promote immune activation or tolerance. One possible strategy to promote mucosal T cell effector and memory immune responses is to conjugate *rS*. *mitis* to charge-switching synthetic adjuvant nanoparticles (cSAPs). cSAP conjugated to inactivated *Chlamydia trachomatis* vaccine was recently shown to induce protective memory T cells and prevent Chlamydia infection in mice [37]. Combining r*S*. *mitis* antibody-based vaccines with T cell vaccines to achieve both antibody and T cell immunity in mucosal and systemic compartments would also be an attractive vaccine regimen.

It is entirely possible that the T cell non-responsiveness observed in *rS*. *mitis* orally vaccinated mice was not be due to the mechanism of immunologic tolerance. A plausible alternative explanation is that mucosal surface colonization by *rS*. *mitis* promotes the trafficking of antigen-specific T cells from the periphery to the oral and gut mucosa [38]. This possibility is now being explored experimentally.

We have generated *rS*. *mitis* vaccine vector as a potential vaccine against mucosal pathogens such as HIV-1. As a pioneer oral commensal bacterium, *S*. *mitis* should be safe as a mucosal vaccine vector for infants, children and adults. The ability of *rS*. *mitis* to induce systemic and mucosal antibody responses warrants further development of *rS*. *mitis* strategy against HIV and other mucosal pathogens. If *S*. *mitis* is proven to be capable of inducing T cell tolerance, *S*. *mitis*-based immunotherapy could also be developed to help prevent the progression of inflammatory diseases as well as allergic and autoimmune diseases.

## Supporting Information

S1 Fig Anti-HIV antibody response in conventional mice after repeated immunization with recombinant *S*. *mitis*. Conventional specific pathogen free (SPF) mice were inoculated with 10^9^ cfu recombinant *S*. *mitis* 3 consecutive days on a weekly basis for a total of 4 weeks. IgA, IgG1, and IgG2a antibody responses in the saliva and serum were assessed on the indicated days following the last inoculation. The mean optical density (O.D) values ± SEM of the antibody produce in the undiluted samples are shown.
(PPTX)
