# Correction: Oral Delivery of a Novel Recombinant *Streptococcus mitis* Vector Elicits Robust Vaccine Antigen-Specific Oral Mucosal and Systemic Antibody Responses and T Cell Tolerance

**DOI:** 10.1371/journal.pone.0147781

**Published:** 2016-01-22

**Authors:** Emily Xie, Abhiroop Kotha, Tracy Biaco, Nikita Sedani, Jonathan Zou, Phillip Stashenko, Margaret J. Duncan, Antonio Campos-Neto, Mark J. Cayabyab

Fig 2 is incorrect. Panel D is missing. The authors have provided a corrected version here.

**Fig 2:**
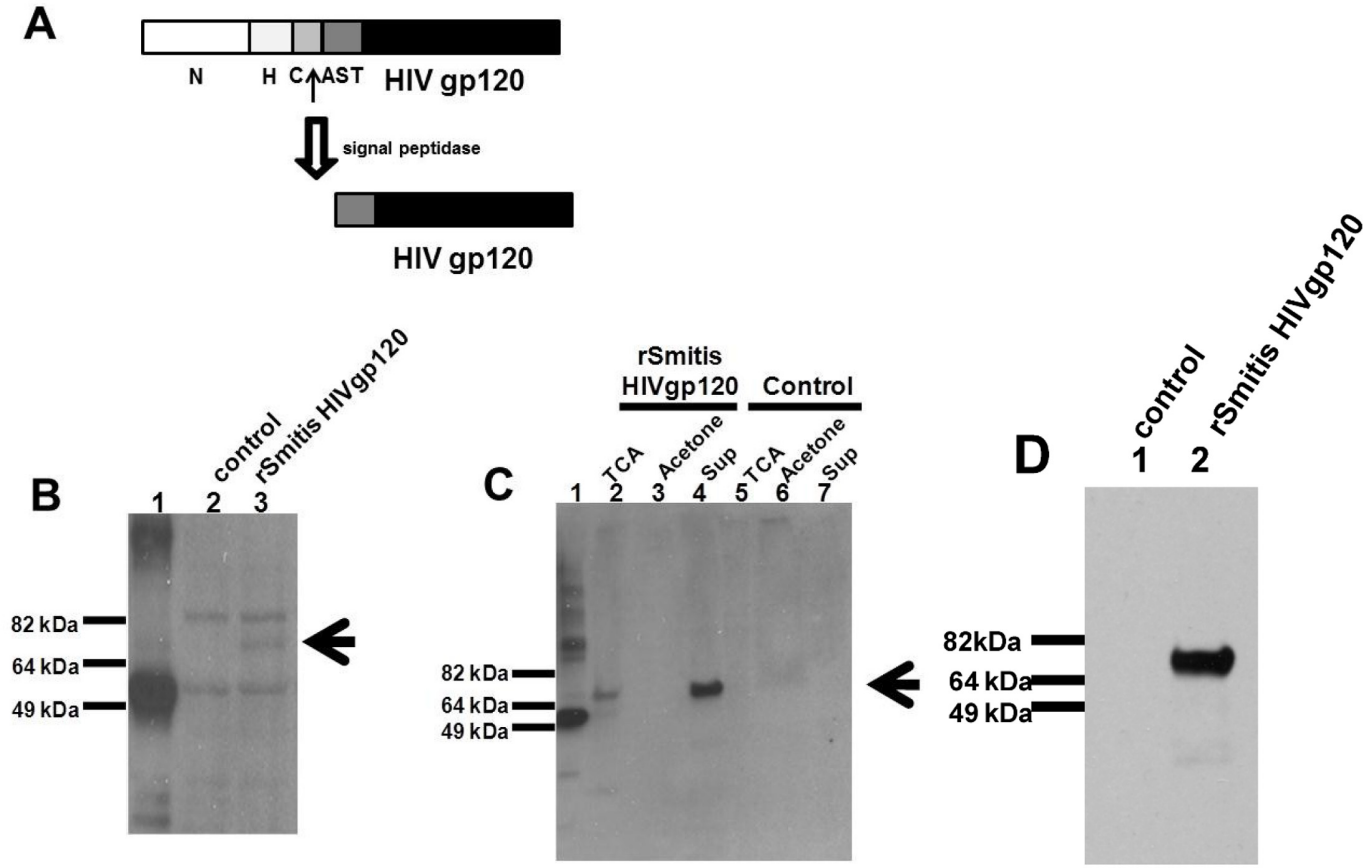
Recombinant *S*. *mitis* expresses HIV envelope protein. *rS*. *mitis* with the integrated HIV HXBc2 Env gp120 was designed to secrete HIV Env by ligating the HIV Env in frame with 250bp 5’ end of the pullulanase gene (pulA/Smt0163) encoding a signal peptide that allows processing and secretion of the HIV antigen. (A) The signal peptide has an amino-terminal region (N), a hydrophobic core (H), a signal peptidase cleavage site (C), and an accessory Sec transport motif (AST). Expression of HIV Env containing a C-terminal His tag was assessed by Western blotting using Penta-His-HRP from a representative recombinant clone in *S*. *mitis* lysates (B) and in culture supernatants (C) by TCA-precipitation (TCA), acetone precipitation (Acetone) and Amicon filter-concentration (Sup). HIV Env expression in lysates (B) and supernatants of control S. mitis vector (control) (C) is shown. The arrow denotes expression of the Env Ag band. 100 ng of His-tagged *M*. *tuberculosis* protein (MT0401) was used as a positive control (B and C, lane 1). (D) The expression of HIV-1 gp120 in *rS*. *mitis* containing the HIV Env gene (lane 2) in Amicon filter-concentrated supernatant was detected using human HIV patient sera. *rS*. *mitis* containing the empty plasmid was used as a negative control (lane 1).
