# Supplementary figures and images for: Oral Delivery of a Novel Recombinant Streptococcus mitis Vector Elicits Robust Vaccine Antigen-Specific Oral Mucosal and Systemic Antibody Responses and T Cell Tolerance
Source: PLoS One. 2015 Nov 30;10(11):e0143422. doi: 10.1371/journal.pone.0143422 (PMC4664415; doi:10.1371/journal.pone.0143422)

## Slide 1
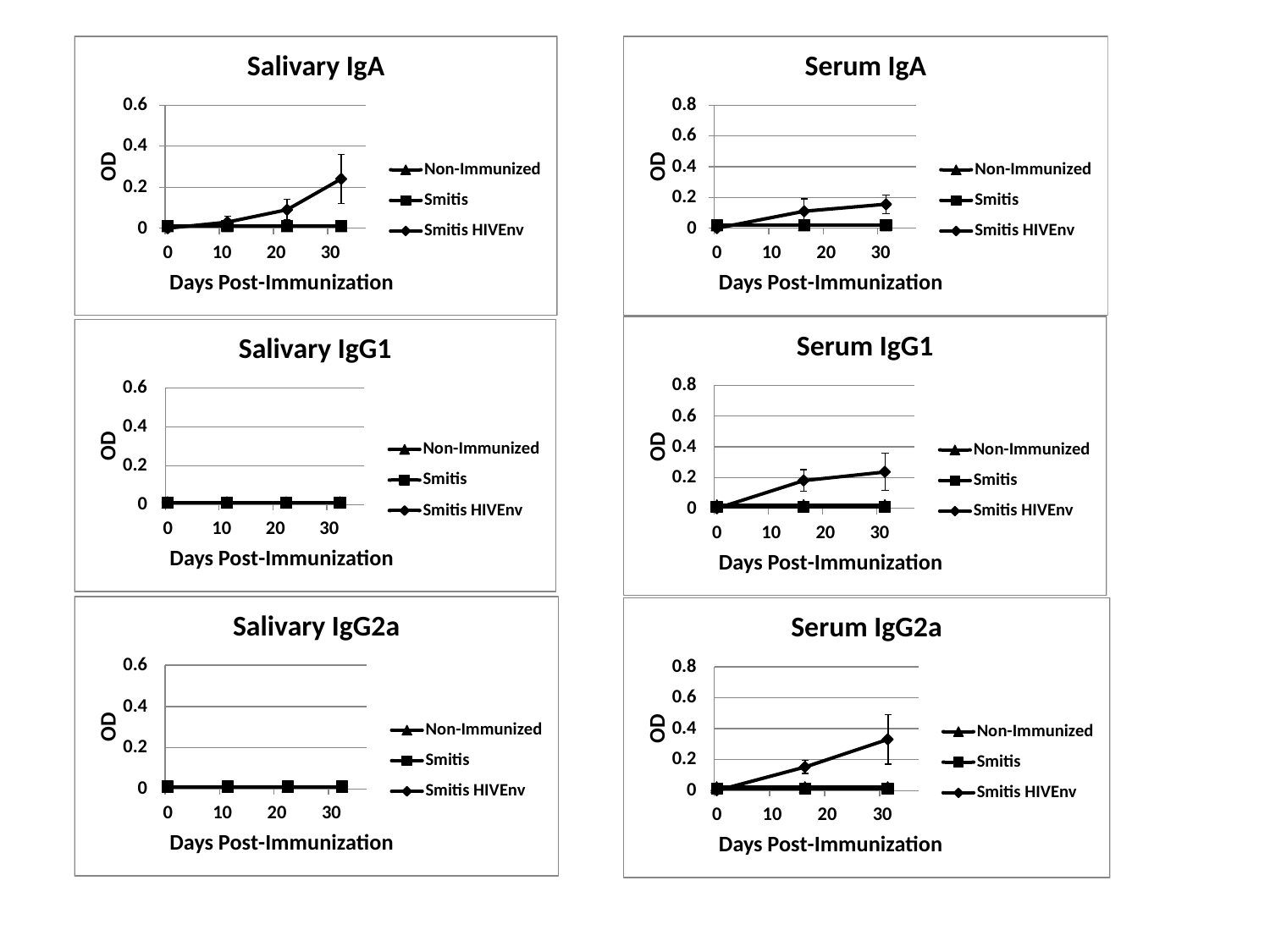

Supplement: S1 Fig — Conventional specific pathogen free (SPF) mice were inoculated with 109 cfu recombinant S. mitis 3 consecutive days on a weekly basis for a total of 4 weeks. IgA, IgG1, and IgG2a antibody responses in the saliva and serum were assessed on the indicated days following the last inoculation. The mean optical density (O.D) values ± SEM of the antibody produce in the undiluted samples are shown. (PPTX) [file pone.0143422.s001.pptx]
